
JOURNAL INFORMATION
==============================
NLM Title Abbreviation: Wirtschaftsdienst
Journal ID: Wirtschaftsdienst
NLM Journal ID: 101086612

Wirtschaftsdienst (Hamburg, Germany : 1949)

ISSN: 0043-6275

ARTICLE INFORMATION
==============================
PMCID: PMC8452498
PMID: 34565924
DOI: 10.1007/s10273-021-3010-6
Article ID: 3010
Article version: 1
Subjects: Ökonomische Trends

Konjunkturschlaglicht: COVID-19 und die Wirtschaft in Afrika

Economic headline: COVID-19 and the economy in Africa

Gern Klaus-Jürgen Klaus-Juergen.Gern@ifw-kiel.de
1
Meuchelböck Saskia Saskia.Meuchelboeck@ifw-kiel.de
2
1 grid.462465.7 0000 0004 0493 2817 Forschungsgruppe Intern. Konjunktur, Institut für Weltwirtschaft (IfW), Kiellinie 66, 24105 Kiel, Deutschland
2 grid.462465.7 0000 0004 0493 2817 Institut für Weltwirtschaft (IfW), Kiellinie 66, 24105 Kiel, Deutschland
Electronic publication date: 2021 Sep 21
Print publication date: 2021
Volume: 101
Issue: 9
First page: 743
Last page: 744
Copyright: © Der/die Autor:in 2021
License: Open Access: Dieser Artikel wird unter der Creative Commons Namensnennung 4.0 International Lizenz veröffentlicht (https://creativecommons.org/licenses/by/4.0/deed.de).
Open Access wird durch die ZBW — Leibniz-Informationszentrum Wirtschaft gefördert.
License URL: https://creativecommons.org/licenses/by/4.0/

issue-copyright-statement: © The Author(s) 2021

==============================

Literatur

Bloomberg (2021), One In Four People In Africa’s Biggest City May Have Had Covid, 23. Februar.
Gern, K.-J., O. Lück und S. Meuchelböck (i.E.), Covid-19 in Africa and its impact on the economy, Kiel Policy Brief, im Erscheinen.
Google (2021), Covid-19 Mobility Report (Workplace mobility), https://www.google.com/covid19/mobility/ (8. September 2021).
IMF (International Monetary Fund) (2021), Regional Economic Outlook. Sub-Saharan Africa — Navigating a Long Pandemic, April.
Mahler, D., N. et al. (2021), Updated estimates of the impact of COVID-19 on global poverty: Turning the corner on the pandemic in 2021?, World Bank Blogs, 6. September.
Tessema S Nkengasong J Understanding COVID-19 in Africa Nature Reviews Immunology 2021 21 469 470 10.1038/s41577-021-00579-y PMC8222946 34168345
The Economist (2021), The pandemic’s true death toll, 2. September.
WHO (World Health Organization) (2021), Eight in 10 African countries to miss crucial COVID-19 vaccination goal, 2. September.
Weltbank (2021), World Development Indicators.
